# Maxillary Arch Expansion and Adenotonsillectomy in Prepubertal Children Diagnosed with Paediatric Obstructive Sleep Apnoea: An Interventional Study

**DOI:** 10.3390/jcm15082981

**Published:** 2026-04-14

**Authors:** Derek Mahony, Niroj Bhattarai, Peter Petocz

**Affiliations:** 1 Department of Orthodontics and Pediatric Dentistry, Faculty of Dentistry, University of Szeged, Tisza Lajos Krt. 64–66., 6720 Szeged, Hungary; derek.mahony@fullfaceorthodontics.com.au; 2 Private Practice, Full Face Orthodontics Inner West, Haberfield, NSW 2045, Australia; 3 Conservatorium of Music, The University of Sydney, Sydney, NSW 2006, Australia; peter.petocz@mq.edu.au

**Keywords:** paediatric, obstructive sleep apnoea (OSA), adenotonsillectomy, semi-rapid maxillary expansion (SRME), respiratory disturbance index (RDI), craniofacial morphology, multidisciplinary treatment, polysomnography (PSG)

## Abstract

**Background:** In many children aged 7–9 years diagnosed with obstructive sleep apnoea (OSA) the decision of which treatment to perform still remains unclear. This is particularly relevant when the children have both a narrow maxilla and enlargement of tonsils and adenoids. Current guidelines recommend adenotonsillectomy (TA) as first-line therapy, but residual OSA is common, prompting interest in adjunctive semi-rapid maxillary expansion (SRME). This study evaluated the effects of TA and SRME on the respiratory disturbance index (RDI) in prepubertal children with OSA, both individually and in combination, regardless of treatment sequence. **Materials and Methods:** In this retrospective cohort study, 80 children (aged 7–9 years) with polysomnography-confirmed obstructive sleep apnoea, narrow maxillary arches, and adenotonsillar enlargement underwent TA first followed by SRME (*n* = 39) or SRME first followed by TA (*n* = 41). Level 1 polysomnography was performed at baseline and 3 months after each intervention. Repeated-measures analyses investigated the RDI profiles of the two groups over time, in each case adjusting for relevant background variables. **Results:** Baseline mean RDI was 18.99 ± 1.66 events/hour. Adjusted for background variables (including, most importantly, BMI), the initial reduction was significantly greater after SRME than TA (adjusted mean difference 1.49 events/hour, *p* = 0.002), and this difference persisted until after both treatments were applied (adjusted mean difference 1.42, *p* = 0.007). **Conclusions:** Combined TA and SRME produced substantial RDI reductions compared to individual interventions in children with dual soft-tissue and skeletal OSA contributors, with treatment order (as well as BMI) significantly associated with different final outcomes. These findings support a flexible, multidisciplinary approach to optimise airway management and reduce residual disease.

## 1. Introduction

Paediatric obstructive sleep apnoea (OSA) is a prevalent form of sleep-disordered breathing characterised by recurrent episodes of partial or complete upper airway obstruction during sleep, leading to intermittent hypoxaemia, hypercapnia, sleep fragmentation, and disruption of normal sleep architecture [1]. Overnight, in-laboratory polysomnography remains the gold standard [2] for diagnosis, with disease severity commonly quantified using the apnoea-hypopnoea index (AHI) or respiratory disturbance index (RDI) [3]. The RDI includes apnoeas, hypopnoeas, and respiratory effort-related arousals (RERAs). Children’s higher respiratory rate and lower lung capacities allow shorter respiratory events (lasting at least two breaths) to have major physiological consequences, making them clinically meaningful, in contrast to adults, where respiratory events must last ≥10 s [4]. The AHI classifies the severity of OSA into three categories: mild (1–4), moderate (5–9), and severe (≥10). One episode of apnoea or hypopnoea per hour in children is regarded as pathological [5]. RDIs are slightly higher than the AHI as they include additional events.

Recent epidemiological studies indicate that the prevalence of paediatric OSA is increasing worldwide, with reported rates approaching 9.5–11% [6]. This rising burden reflects changing risk profiles in children, and highlights the need for effective, durable treatment strategies.

If left untreated, paediatric OSA is associated with significant short- and long-term morbidity, including impaired neurocognitive development, behavioural disturbances such as attention-deficit/hyperactivity disorder, poor academic performance, cardiovascular and metabolic dysfunction, growth impairment, and reduced quality of life [7,8,9,10,11]. Early identification and appropriate intervention are, therefore, critical during periods of active growth and neurodevelopment [12].

Adenotonsillar hypertrophy is widely recognised as the primary aetiological factor in paediatric OSA [13,14,15,16,17]. Enlarged adenoids and tonsils narrow the upper airway, increasing resistance and collapsibility during sleep, leading to recurrent obstruction, hypoxaemia, and sleep fragmentation [18]. Consequently, current clinical guidelines internationally recommend adenotonsillectomy as first-line therapy in most children [19,20,21,22]. Although adenotonsillectomy results in significant improvements in respiratory indices and symptoms, large multicentre and longitudinal studies have demonstrated that a substantial proportion of children exhibit residual or recurrent OSA following surgery, particularly older children and those with obesity [21,23,24]. Increasing attention has, therefore, been directed toward non-lymphoid contributors to persistent airway obstruction, particularly craniofacial morphology. Systematic reviews and morphometric studies have consistently demonstrated that children with OSA frequently present with maxillary constriction, high and narrow palatal vaults, mandibular retrognathia, and hyperdivergent growth patterns [25]. These skeletal features reduce nasal and pharyngeal airway volume, posteriorly displace the tongue, and promote mouth breathing, thereby exacerbating airway obstruction [26,27].

While most clinical studies and systematic reviews have supported the efficacy of maxillary expansion in the management of paediatric OSA [28,29,30], a recent systematic review and network meta-analysis did not demonstrate significant reductions in apnoea–hypopnoea indices, or improvements in oxygen saturation, following expansion alone [31]. Importantly, multidisciplinary studies comparing adenotonsillectomy and maxillary expansion have shown that both interventions can independently improve OSA outcomes, with similar benefits observed regardless of whether surgical or orthodontic treatment is performed first [32]. These findings underscore the rationale for a treatment paradigm that concurrently addresses both soft tissue and skeletal contributors to airway obstruction.

Given the increasing prevalence of paediatric OSA, the substantial rates of residual disease following adenotonsillectomy, and the strong association between craniofacial morphology and airway function, a multidisciplinary approach to management is warranted. The present study aims to evaluate the combined effects of adenotonsillectomy and maxillary arch expansion in prepubertal children with OSA, assessing improvements in respiratory indices, and clinical outcomes, to inform optimised, evidence-based treatment pathways.

## 2. Materials and Methods

### 2.1. Study Design

This retrospective cohort study was conducted as a follow-up analysis on a subsample of patients from a larger cohort, previously described in a study evaluating the association between craniofacial patterns and obstructive sleep apnoea (OSA) in children seeking orthodontic treatment [25]. The current study aimed to assess the impact of two interventions—semi-rapid maxillary expansion (SRME) and adenotonsillectomy (TA)—on the respiratory disturbance index (RDI), both individually and in combination, regardless of treatment order. Ethical approval was obtained from the medical ethics committee of the University of Greater Manchester, and informed consent had been previously obtained from parents or guardians during initial clinical care. The study adhered to the principles outlined in the Declaration of Helsinki.

### 2.2. Sample Selection

**Figure 1:**
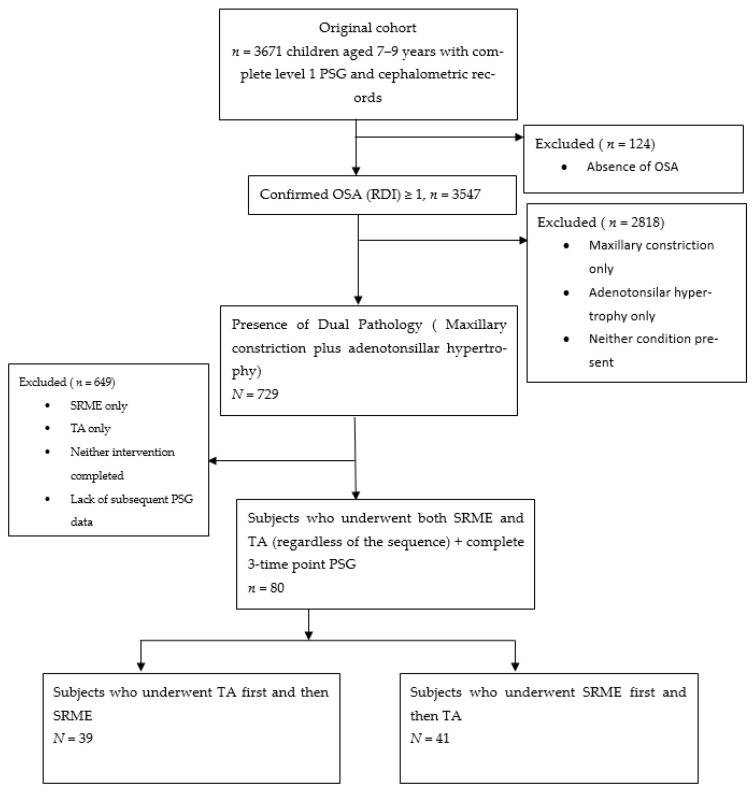
Patient selection flow diagram showing derivation of the final analytical sample of 80 children from the original cohort of 3671 children who had complete polysomnographic and cephalometric records [23]. Numbers and specific reasons for exclusion at each stage are shown.

From the original cohort of 3671 children, aged 7 to 9 years, who underwent polysomnography (PSG) and cephalometric analysis during their first orthodontic consultation (between February 2007 and December 2022 [25]), a subsample of 80 patients was identified for inclusion in this study (Figure 1). These patients were selected based on the following criteria:

- Availability of complete PSG records at three time points: baseline (pre-treatment), post-first intervention, and post-both interventions.
- Completion of both SRME and TA interventions, regardless of sequence.
- No history of prior orthodontic treatment, ENT surgery, or diagnosed sleep-disordered breathing (SDB) at baseline.
- Baseline RDI ≥ 1 (indicating at least mild OSA), as determined from the initial PSG.
- Aged 7–9 years at baseline, with no genetic syndromes, craniofacial abnormalities, developmental issues, maxillofacial trauma, or tumour history.

Exclusion criteria mirrored the original study, including incomplete records, home-based sleep studies (only Level 1 hospital-based PSGs were included), or non-completion of both interventions.

The subsample was divided into two groups based on treatment sequence, determined by patient/parental preference, and logistical factors, rather than randomization:

1. **Group 1 (TA first; *n* = 39)**: Patients who underwent TA as the initial intervention, followed by SRME (ENT first in data sheet terminology).
2. **Group 2 (expansion first; *n* = 41)**: Patients who underwent SRME as the initial intervention due to delays in accessing public ENT appointments, or waiting periods for private insurance coverage for TA (EXP first in data sheet terminology).

Treatment sequences were not randomised but reflected real-world clinical decision-making. The various background variables that could have impacted results—Sex (M or F), Race (Caucasian, East Asian, South Asian or Other), BMI (UH underweight or healthy, OW overweight, OB obese), Age (grouped into tertiles, 7.0–7.9, 8.0–8.6, 8.7–9.0 years)—were adjusted for in the statistical modelling.

### 2.3. Interventions

All interventions were performed in the original clinical settings (nine private orthodontic clinics for SRME and accredited ENT facilities for TA).

**Figure 2:**
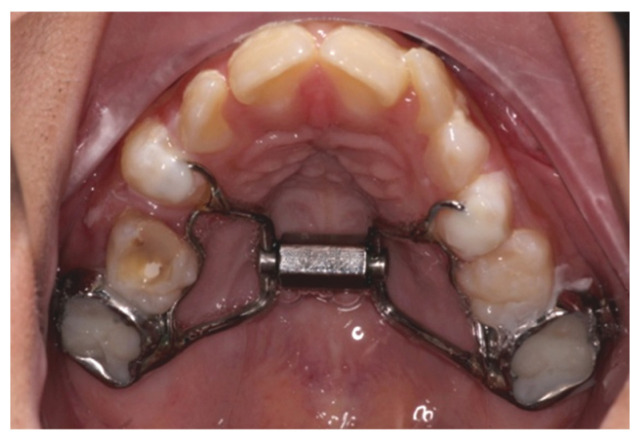
Semi-rapid maxillary expansion (SRME) using a banded hyrax.

1. **Semi-rapid maxillary expansion (SRME)**: SRME differs from conventional RME in its slower activation protocol (1/8 mm per turn daily, approximately 0.125–0.25 mm/day) compared with conventional RME (typically 0.5–1 mm/day). A banded hyrax (superscrew design) expander was used to achieve transverse maxillary expansion. The activation protocol involved 1/8 mm per turn, daily, until the desired expansion (typically 5–8 mm, based on clinical assessment of palatal suture opening and resolution of crossbite or arch constriction). This gradual approach is intended to promote more physiological sutural remodelling and reduce the risk of relapse and discomfort. The protocol was standardised across all nine participating private orthodontic clinics. The expansion duration averaged 8–10 weeks, followed by a 3-month retention period with the appliance in place. Compliance and correct activation were monitored via follow-up orthodontic visits (Figure 2). Mid-palatal suture opening was assessed clinically rather than radiographically to minimise radiation exposure in this paediatric population.

2. **Adenotonsillectomy (TA)**: Surgical removal of tonsils and adenoids was performed by board-certified otolaryngologists (ENTs) under general anaesthesia, in hospital settings. Indications included adenotonsillar hypertrophy confirmed by clinical examination and/or endoscopy, with grading per Brodsky scale (tonsils) and Friedman scale for adenoid size assessment. Postoperative recovery was monitored, with no complications reported in this subsample.

Post-first intervention PSGs were conducted 3 months after the initial treatment to allow for healing and stabilisation. Final PSGs (after both interventions) were performed 3 months after the second treatment.

### 2.4. Data Collection

Baseline data were derived from the original study records, including demographic details, BEARS questionnaire scores (≥5 indicating SDB risk), and cephalometric classifications.

#### Polysomnography (PSG) Measurements

All PSGs were Level 1 in-laboratory studies conducted at accredited paediatric sleep centres, following the Australasian Sleep Association guidelines [24]. Respiratory events were manually scored by certified sleep technicians, blinded to treatment sequence. The primary outcome measure was the respiratory disturbance index (RDI), defined as the average number of apnoeas, hypopnoeas, and respiratory effort-related arousals (RERAs) per hour of sleep. Scoring criteria followed the American Academy of Sleep Medicine (AASM) paediatric rules:

1. Apnoea: ≥90% reduction in airflow for ≥2 missed breaths, with continued respiratory effort (obstructive) or absent effort (central).
2. Hypopnoea: ≥30% reduction in airflow for ≥2 missed breaths, associated with ≥3% oxygen desaturation or arousal.
3. RERA: Sequence of breaths with increasing respiratory effort leading to arousal, without meeting apnoea/hypopnoea criteria.

The RDI was calculated at baseline, post-first intervention, and post-both interventions.

### 2.5. Statistical Analysis

Statistical analyses were conducted using SPSS version 31.0 (IBM Corp., Armonk, NY, USA). A repeated-measures analysis of variance was carried out for the RDI profiles over time, using Order, Sex, Race, BMI and Age group as factors, as well as BMI by Order interaction. The statistical assumptions required for this model were checked and found to be satisfied. Statistical significance was set at *p* < 0.05, with values between 0.01 and 0.05 treated as ‘marginally significant’. The Greenhouse–Geisser adjustment was applied to correct for non-sphericity.

## 3. Results

### 3.1. Sample Characteristics

The study sample comprised records of 80 children aged 7 to 9 years (mean age 8.31 ± 0.58 years, range 7.0–9.0 years) out of which 35 (43.8%) were males and 45 (56.3%) were females. The mean RDI was 18.99 ± 1.66 events per hour across the entire sample. Participants were divided into two groups according to the sequence of interventions. The first group (*n* = 39) underwent TA first, followed by SRME. The second group (*n* = 41) received SRME first, followed by TA. Baseline demographic and clinical characteristics are present in Table 1.

### 3.2. Adjusted RDI Values Post Intervention

The repeated-measures analysis of variance showed that Order had a significant effect on the RDI profiles over time (*p* = 0.001) as well as Order by BMI interaction (*p* = 0.009), with the main effect of BMI not significant (*p* = 0.092). The other background variables were not significant (Sex *p* = 0.92, Race *p* = 0.39, Age group *p* = 0.90). The table below (Table 2) shows the estimated (model-based) mean and standard error of RDI values at each of the time points (1 = pre, 2 = mid, 3 = final) for each of the BMI categories (HU = healthy/underweight <85 percentile, OW = overweight 85 to <95 percentile, OB = obese 95 percentile and above).

**Figure 3:**
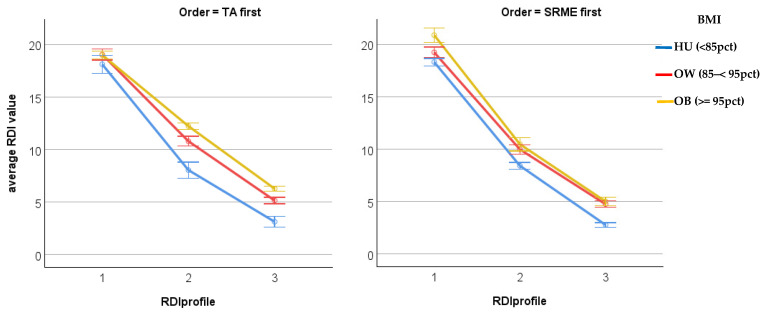
Plot of adjusted RDI values by Order, BMI and Time. Error bars +/− 1 SE.

The results are summarised graphically in Figure 3 above; both groups (TA first and SRME first) show a dramatic decline from the baseline RDI to the end of the first period and this decline continues with only a slightly lower rate to the end of the second period. Generally, the RDI values in Group 2 (SRME first) end up lower than those in Group 1 (TA first), though there is an indication that baseline values in Group 1 are lower on average than those in Group 2. There is a strong indication that the HU (healthy/underweight) group has lower RDI values—even initially—and the OB (obese) group remains with the highest RDI values. The significant Order by BMI interaction is shown by the fact that in each group the three coloured lines are not (quite) parallel.

### 3.3. Adjusted RDI Reductions and Percentage Reductions Post Intervention

The statistical model shows that RDI reductions overall from baseline (time 1) to post-first treatment (time 2) and from baseline to post intervention (time 3) were statistically significant (*p* < 0.001 in all cases). However, the actual RDI reductions and percentage reductions are dependent on Order of treatment and BMI grouping. Table 3 (below) shows the RDI reductions and percentages for each Order by BMI grouping.

Adjusted for background variables, the reduction post-first treatment was significantly greater for Group 2 (after SRME, 50.6%) than for Group 1 (after TA, 44.6%) with an adjusted mean difference of 1.49 events per hour (*p* = 0.002). This difference persisted until after both treatments, with a 78.5% reduction in Group 2 and 74.1% reduction in Group 1, with an adjusted mean difference of 1.42 events per hour (*p* = 0.007). However, these results are for the BMI groups combined. The table shows nuanced differences for separate BMI groups: for the healthy or underweight BMIs (up to the 85th percentile) the differences are negligible, while they are greater for the overweight group (BMIs from the 85th up to the 95th percentiles). The greatest differences, over three events per hour, occur in the OB group, with BMIs at or exceeding the 95th percentile.

## 4. Discussion

This retrospective cohort study evaluated 80 prepubertal children aged 7–9 years with polysomnography-confirmed obstructive sleep apnoea, a constricted maxillary complex, and adenotonsillar hypertrophy. Statistically and clinically significant reductions in the RDI were observed following TA and SRME, whether performed as standalone interventions or in combination. When TA was performed as the initial treatment, the adjusted reduction in RDI was 8.36 events/hour (approximately 44.6%), whereas initiation with SRME resulted in a greater adjusted reduction of 9.85 events/hour (approximately 50.6%). The combined treatment approach yielded the largest improvement, with an RDI reduction of 13.87 events/hour (approximately 74.1%) in the TA-SRME sequence, whereas an RDI reduction of 15.29 events/hour (approximately 78.5%) was achieved in the SRME-TA sequence. The final adjusted RDI of 4.86 and 4.19 events/h in the respective sequences falls in the mild residual OSA range. Complete normalisation (RDI < 1) was not achieved in all participants; however, the reduction from a baseline mean of 18.99 events/h represents a substantial clinical improvement.

SRME produced clinically meaningful reductions in the RDI when used as the initial intervention. This effect is consistent with the biomechanical changes induced by RME, including mid-palatal suture separation, decreased nasal airway resistance, increased nasal cavity volume, and secondary enlargement of the pharyngeal airway space. These structural modifications facilitate nasal breathing, improve tongue posture, and reduce upper airway collapsibility during sleep [33]. The choice of SRME over RME is based on the evidence that SRME slowly remodels the palate reducing the risk of relapse, allowing it to flatten and improve the airway volume [34,35]. Systematic reviews and meta-analyses have consistently demonstrated that maxillary expansion is associated with reductions in the apnoea–hypopnoea index and improvements in oxygen saturation in children with transverse maxillary deficiency and paediatric obstructive sleep apnoea [36,37]. Additional supporting evidence includes high rates of transition from mouth to nasal breathing [38], increased sagittal upper airway dimensions and counterclockwise mandibular rotation [39], alleviation of snoring and daytime sleepiness [40], and repeated documentation of narrower maxillary arches, higher palatal vaults, and reduced palatal support tissues in mouth-breathing children compared with nasal breathers [27,41,42]. These findings collectively support the application of SRME in prepubertal children with craniofacial features contributing to OSA.

The presence of residual RDI following SRME is consistent with the meta-analysis findings of Quinzi et al. [28], who reported a post-RME apnoea–hypopnoea index (AHI) of 2.5 ± 2.6 events/hour across 102 children, indicating that standalone SRME may not fully resolve OSA [28]. Potential contributing factors include uncorrected adenotonsillar hypertrophy (which SRME may indirectly reduce but does not eliminate), persistent nasal or pharyngeal soft-tissue resistance, incomplete craniofacial remodelling in some cases, or coexisting factors such as allergic rhinitis or neuromuscular tone deficits. In contrast, Cistulli et al. [43] demonstrated a marked reduction in the apnoea–hypopnoea index (AHI) from 19 ± 4 to 7 ± 4 events/hour in a group of adult patients undergoing maxillary expansion, with seven of the 10 participants achieving normalisation of the AHI postoperatively [43]. It is worth noting that the literature on maxillary expansion for paediatric OSA shows some variability. A recent pilot study by Colonna et al., showed no statistically significant outcome following RME [44]

Adenotonsillectomy (TA) as the initial intervention also resulted in substantial RDI improvement. This is in keeping with its established role as the primary treatment for adenotonsillar hypertrophy, the most common anatomical cause of paediatric obstructive sleep apnoea [45]. Despite a significant improvement, there was incomplete resolution which is consistent with large multicentre studies reporting residual disease in 20–40% of otherwise healthy children following TA alone, with rates approaching or exceeding 49% in obese subgroups [46,47,48].

Potential contributing factors include uncorrected underlying craniofacial abnormalities—such as maxillary constriction, increased palatal height, mandibular retropositioning, or small mandibular size [49]—ongoing nasal obstruction from allergic rhinitis or chronic adenoidal remnants, persistent mouth breathing, and other unaddressed contributors such as neuromuscular tone deficits or obesity-related pharyngeal fat deposition [50]. These observations highlight that, while TA effectively targets the primary soft-tissue obstruction, it does not address skeletal and nasal factors that may sustain airway compromise in children with combined aetiologies, thereby supporting the addition of SRME to achieve more complete normalisation of respiratory indices.

The combination of TA and SRME yielded the greatest overall RDI reduction, with final values reduced by 66–85%; the SRME-TA order was associated with modestly greater adjusted RDI reductions compared with the TA-SRME order particularly for the subjects with a BMI in the OW and especially OB range. This finding differs from the pilot findings of Guilleminault et al. [32], who observed comparable polysomnographic improvements after crossover in prepubertal children with narrow maxillae and moderate tonsillar enlargement, regardless of whether adenotonsillectomy or RME was initiated first [32]. The superior cumulative effect most likely reflects the complementary nature of the two interventions: TA directly removes the dominant soft-tissue obstruction, while SRME corrects the underlying skeletal and nasal deficiencies that may limit the efficacy of TA or predispose to recurrence. Longitudinal data from Villa et al. similarly indicate that SRME serves as an effective adjunct in children with moderate adenotonsillar hypertrophy, with sustained benefits evident years after treatment and an implication that early orthodontic intervention enhances long-term outcomes [51,52].

In this matched retrospective cohort, SRME as the initial intervention was associated with a greater early adjusted reduction in the RDI compared with TA performed first. This differential early response highlights the particular potency of SRME in this population, where transverse maxillary deficiency appears to play a prominent role in sustaining airway compromise. Evidence has also shown significant post-expansion volumetric decreases in both adenoid and palatine tonsil tissue post-expansion, which may partly explain why SRME improved OSA better than TA [53].

In contrast, while TA effectively targets the dominant soft-tissue obstruction in many cases, its initial impact in this craniofacially compromised subgroup was comparatively more modest, likely reflecting the persistence of underlying skeletal and nasal factors that SRME addresses more directly [48]. These observations suggest that, in children presenting with both adenotonsillar hypertrophy and a constricted maxillary complex, initiating treatment with SRME may be associated with a greater early reduction in RDI in this cohort, by tackling a key structural contributor to airway obstruction before surgical removal of lymphoid tissue.

This retrospective cohort study benefits from objective Level 1 polysomnography assessments at three time points and a pragmatic design reflecting real-world clinical decision-making, which enhances external validity. The focus on a narrow prepubertal age range (7–9 years) during active craniofacial growth further strengthens relevance for interventions such as SRME. However, the retrospective and non-randomised design is an important limitation. Treatment sequence was determined by logistical factors rather than random allocation, raising the possibility of residual allocation bias despite multivariate adjustment through repeated-measures ANOVA that included the BMI category and the Order × BMI interaction. Additional limitations include the relatively short follow-up period of three months after each intervention. Orthodontic stability and airway remodelling are long-term processes, and the durability of the observed RDI improvements, as well as the risk of recurrence, remain unknown. Moreover, the private-practice cohort may reduce generalisability to broader populations or public health systems. 

These findings support early consideration of SRME in prepubertal children with OSA and maxillary constriction, as it may provide a greater initial reduction in respiratory events than TA alone. The broadly comparable final outcomes (a difference of only 1.42 events per hour) irrespective of treatment sequence support a flexible, multidisciplinary approach guided by clinical factors and logistics, with routine orthodontic assessment of transverse maxillary dimensions, potentially improving selection for combined therapy and reducing residual disease during critical growth periods.

Despite combined surgical and orthodontic intervention, complete normalisation of RDI was not achieved, underscoring the multifactorial nature of paediatric OSA and the contribution of persistent nasal resistance, altered tongue posture, and neuromuscular factors beyond lymphoid and skeletal obstruction. Adjunctive therapies such as orofacial myofunctional therapy may, therefore, be required to further optimise residual disease, in selected cases.

Prospective randomised controlled trials incorporating long-term follow-up, volumetric airway imaging, and stratification by baseline severity, obesity, and craniofacial phenotype are warranted to validate these findings, refine treatment sequencing, and assess long-term effectiveness and cost-efficiency.

## 5. Conclusions

Both TA and SRME significantly improved the RDI in prepubertal children with OSA and maxillary constriction, with the greatest benefit observed when combined. The SRME-TA sequence was somewhat more effective than the reverse order, by about 1.5 events/hour overall, but increasing to around three events/hour in the obese group with the highest 5% BMIs. Expansion as the initial intervention in this cohort produced a larger early reduction in the RDI than TA alone, highlighting the potential value of addressing transverse maxillary deficiency early in children with evident skeletal contributor. Despite combined therapy, complete normalisation was not consistently achieved, reflecting the multifactorial nature of paediatric OSA. Future prospective studies are needed to refine treatment sequencing, assess long-term durability and clinical outcomes, and explore adjunctive strategies for residual disease.

## Figures and Tables

**Table 1:** Demographic characteristics of sample.

Characteristics	Overall (*N* = 80)	Group 1 TA First (*n* = 39)	Group 2 SRME First (*n* = 41)
Mean Age	8.31 ± 0.58	8.34 ± 0.55	8.28 ± 0.62
Baseline RDI (Mean)	18.99 ± 1.66	18.97 ± 1.65	19.00 ± 1.69
Male	35	18	17
Female	45	21	24
BMI group: HU (<85 pct)	27	4	23
OW (85 to <95 pct)	23	11	12
OB (≥95 pct)	30	24	6

**Table 2:** Adjusted RDI values by Order, BMI and Time (estimated from the statistical model).

Order	BMI	Time	Mean RDI	SE	Order	BMI	Time	Mean RDI	SE
TA first	HU	1	18.12	0.85	SRME first	HU	1	18.32	0.35
(Group 1)		2	8.05	0.76	(Group 2)		2	8.43	0.32
		3	3.14	0.52			3	2.79	0.22
	OW	1	19.05	0.52		OW	1	19.25	0.50
		2	10.80	0.47			2	9.99	0.45
		3	5.16	0.32			3	4.76	0.31
	OB	1	19.00	0.36		OB	1	20.88	0.70
		2	12.24	0.33			2	10.49	0.63
		3	6.28	0.22			3	5.02	0.43
	(Comb)	1	18.73	0.37		(Comb)	1	19.48	0.34
		2	10.36	0.33			2	9.64	0.30
		3	4.86	0.22			3	4.19	0.20

**Table 3:** Adjusted RDI reductions by Order, BMI and Time (estimated from the statistical model).

Order	BMI	Time	RDI Reduction	% Reduction	Order	BMI	Time	RDI Reduction	% Reduction
TA first	HU	1	(18.12)		SRME first	HU	1	(18.32)	
(Group 1)		2	10.07	55.6	(Group 2)		2	9.89	54.0
		3	14.99	82.7			3	15.53	84.9
	OW	1	(19.05)			OW	1	(19.25)	
		2	8.25	43.3			2	9.26	48.1
		3	13.89	72.9			3	14.48	75.2
	OB	1	(19.00)			OB	1	(20.88)	
		2	6.77	35.6			2	10.40	49.8
		3	12.72	66.9			3	15.86	76.0
	(Comb)	1	(18.73)			(Comb)	1	(19.48)	
		2	8.36	44.6			2	9.85	50.6
		3	13.87	74.1			3	15.29	78.5

## Data Availability

The datasets generated during and/or analysed during the current study are available from the corresponding author upon reasonable request.
